# Transcriptome Reprogramming of CD11b^+^ Bone Marrow Cells by Pancreatic Cancer Extracellular Vesicles

**DOI:** 10.3389/fcell.2020.592518

**Published:** 2020-11-27

**Authors:** Joana Maia, Andreia Hanada Otake, Juliana Poças, Ana Sofia Carvalho, Hans Christian Beck, Ana Magalhães, Rune Matthiesen, Maria Carolina Strano Moraes, Bruno Costa-Silva

**Affiliations:** ^1^Champalimaud Centre for the Unknown, Champalimaud Foundation, Lisbon, Portugal; ^2^Graduate Program in Areas of Basic and Applied Biology, University of Porto, Porto, Portugal; ^3^Center for Translational Research in Oncology, Instituto do Câncer do Estado de São Paulo, Hospital das Clínicas, Faculdade de Medicina da Universidade de São Paulo, São Paulo, Brazil; ^4^i3S – Instituto de Investigação e Inovação em Saúde, Universidade do Porto, Porto, Portugal; ^5^IPATIMUP – Instituto de Patologia e Imunologia Molecular da Universidade do Porto, Porto, Portugal; ^6^Instituto de Ciências Biomédicas Abel Salazar, University of Porto, Porto, Portugal; ^7^Computational and Experimental Biology Group, CEDOC, Chronic Diseases Research Centre, NOVA Medical School, Faculdade de Ciencias Medicas, Universidade NOVA de Lisboa, Lisbon, Portugal; ^8^Centre for Clinical Proteomics, Department of Clinical Biochemistry and Pharmacology, Odense University Hospital, Odense, Denmark

**Keywords:** tumor microenvironment, extracellular vesicles, macrophages, monocytes, metastasis, pancreatic cancer, exosomes, cancer

## Abstract

Pancreatic cancers (PC) are highly metastatic with poor prognosis, mainly due to delayed detection. We previously showed that PC-derived extracellular vesicles (EVs) act on macrophages residing in the liver, eliciting extracellular matrix remodeling in this organ and marked hepatic accumulation of CD11b^+^ bone marrow (BM) cells, which support PC liver metastasis. We here show that PC-EVs also bind to CD11b^+^ BM cells and induce the expansion of this cell population. Transcriptomic characterization of these cells shows that PC-EVs upregulate IgG and IgA genes, which have been linked to the presence of monocytes/macrophages in tumor microenvironments. We also report here the transcriptional downregulation of genes linked to monocyte/macrophage activation, trafficking, and expression of inflammatory molecules. Together, these results show for the first time the existence of a PC–BM communication axis mediated by EVs with a potential role in PC tumor microenvironments.

## Introduction

Pancreatic cancer is the fourth leading cause of cancer-related deaths in the world, displaying a 5-year survival rate of about 6% and a median survival rate of about 6 months. Among pancreatic cancers, pancreatic ductal adenocarcinoma (PC) is the most common type and accounts for more than 90% of cases (Saif, 2011). A combination of factors leads to the poor prognosis of PC, including difficulties in detecting early stage disease, its high metastatic potential, and its resistance to conventional therapies. Current predictions report a worldwide escalation in the incidence of this disease and an over twofold increase in the number of new PC cases, as well as related deaths, by 2030 (Ying et al., 2016; Foucher et al., 2018).

Tumors do not exist as isolated entities, but as complex systemic networks involving cell–cell communication between transformed and non-transformed cells. The milieu created by tumor-associated cells can be composed by both local cells and cells recruited from distant sites, such as the bone marrow (BM) (Bergfeld and DeClerck, 2010), creating a tumor microenvironment thought to be a key modulator of tumor progression by providing either inhibitory or stimulatory growth signals (Bissell and Hines, 2011). In sites distant from the primary tumor, non-tumor cells can also be hijacked in order to prepare the future metastatic sites that support engraftment and survival of metastatic cells (Bergfeld and DeClerck, 2010; Lee and Margolin, 2011). Besides direct cell-to-cell communication, secreted factors play a key role in the interaction among cells. Of these, extracellular vesicles (EVs) have emerged as novel cell–cell communication players in setting up and modifying tumor microenvironments (Record et al., 2014; Maia et al., 2018).

Extracellular vesicles are vesicles released by both prokaryotic and eukaryotic cells, being involved in various physiological and pathological processes (Kalluri and LeBleu, 2020). Microvesicles and exosomes are prevalent types of EVs in biofluids. Microvesicles are generated by the direct outward budding of the plasma membrane, and they range in size from ∼100 to 1,000 nm (Stahl and Raposo, 2019). In contrast, exosomes have an endosomal origin and fall within a size range of ∼30 to 150 nm in diameter (Maia et al., 2018; Stahl and Raposo, 2019). Regardless of their type, EVs can harbor biomolecules such as proteins, DNAs, messenger RNAs, microRNAs, and other RNAs (Nolte-’t Hoen et al., 2012). Due to their cargo and capacity to transfer information both locally and to distant sites, consensus has lately emerged on their role as “signal-transducing agents” (Stahl and Raposo, 2019).

The role of EVs in oncology is currently an active area of research. We have recently described a previously unknown prometastatic circuit in which pancreatic cancer-derived EVs can induce the formation of liver premetastatic niches (LPMN) that foster metastatic development (Costa-Silva et al., 2015; Hoshino et al., 2015). We demonstrated that EVs derived from malignant pancreatic lesions play a key role in LPMN initiation by being specifically taken up by Kupffer cells (KC) in the liver, where they activate fibrotic pathways and promote a pro-inflammatory milieu that ultimately supports metastasis. In particular, we showed that exosomal macrophage migration inhibitory factor (MIF) induces the release of transforming growth factor β (TGF-β) by KCs, which, in turn, promotes activation and fibronectin production by hepatic stellate cells. Fibronectin deposits subsequently promote the arrest of CD11b^+^ BM-derived cells in the liver, completing the formation of the LPMN (Costa-Silva et al., 2015). Although we showed that BM cells are a key component of the LPMN, it is still unknown whether PC-EVs have a potential direct effect in BM cells and in their phenotypes.

Considering that virtually any cell in the body is a potential target for these tumor-derived messages, the identification of novel cellular circuits induced by tumor-derived EVs will help to further elucidate the cellular mechanisms involved in oncologic diseases. In this work, we show that PC-EVs preferentially bind to CD11b^+^ BM cells and induce the expansion of this cell population. Additionally, PC-EVs induce phenotypic changes in CD11b^+^ BM cells with potential relevance to the dynamics of the tumor microenvironments. Together, these results demonstrate the existence of a PC–BM communication axis mediated by EVs with a potential role in PC tumor microenvironments.

## Materials and Methods

### Cells

The C57Bl/6 murine pancreatic adenocarcinoma PAN02 (also identified as Panc02) was purchased from the DTP, DCTD Tumor Repository, NIH. Cells were cultured in RPMI supplemented with 10% fetal bovine serum (FBS, Biowest S181BH-500, Nuaillé, France) and 1% penicillin–streptomycin (Gibco 15-140-122, United States), and maintained at 37°C with 5% CO_2_ levels. For conditioning, cells were cultured in RPMI supplemented with 1% penicillin–streptomycin and 10% EV-depleted FBS. FBS was depleted of bovine EVs by ultracentrifugation at 100,000 *g* for 140 min. For the preparation of the conditioned medium, 1 × 10^6^ PAN02 cells were seeded in 150 mm culture dishes containing 20 ml of medium, and the conditioned medium was collected after 72 h of culture.

### EV Isolation

The EV isolation/purification procedure was performed as previously described (Ferreira et al., 2019). Specifically, the conditioned medium was submitted to two initial centrifugations (10 min, 500 *g* and 20 min, 3,000 *g*) to remove any suspended or dead cells in the medium. To remove large EVs, media was centrifuged (20 min, 12,000 *g*) and the pellet was discarded. The supernatant enriched in small EVs was again centrifuged (2 h 20 min, 100,000 *g*), and the EV-enriched pellet was collected. For sucrose cushion purification, this pellet was resuspended in 14 ml filtered phosphate buffered saline (PBS, Corning 15313581, NY, United States) and added to the top of 4 ml sucrose solution (D2O containing 1.2 g of protease-free sucrose and 96 mg of *Tris* base adjusted to pH 7.4). A new ultracentrifugation was performed (1 h 10 min, 100,000 *g*), after which 4 ml of the sucrose fraction was collected using a 18G needle placed at the bottom of the ultracentrifugation tube (away from the pellet). Finally, 16 ml of PBS was added to the collected sucrose/EV solution and an overnight (16 h, 100,000 *g*) ultracentrifugation was performed. The pellet containing the isolated EVs was resuspended in filtered PBS.

All solutions used (PBS and sucrose cushion) were sterile (0.22 μm membrane-filtered). All centrifugation steps were performed in refrigerated conditions (10°C), and ultracentrifugation was performed with rotors 50.4Ti or 70Ti (Beckman-Coulter, CA, United States).

### EV Labeling

For EV-tracking experiments, purified EVs were fluorescently labeled using PKH67 membrane dye (PKH67GL-1KT, Sigma-Aldrich, Germany). The staining was performed during the isolation protocol according to the manufacturer’s instructions. Briefly, labeling was done before the sucrose cushion step. After the fraction with labeled EVs (4 ml) was collected, the isolation protocol was performed as previously stated.

### EV Characterization

All EV samples were analyzed for particle concentration and size distribution by the NS300 Nanoparticle Tracking Analysis (NTA) system with red laser (638 nm) (NanoSight – Malvern Panalytical, United Kingdom). Samples were prediluted in filtered PBS to achieve a concentration within the range for optimal NTA analysis. Video acquisitions were performed using a camera level of 16 and a threshold between 5 and 7. Five to nine videos of 30 s each were captured per sample. Analysis of particle concentration per milliliter and size distribution were performed with the NTA software v3.4.

Additionally, protein quantification of the EV preparations was assessed by BCA assay (Pierce^TM^ BCA Protein Assay kit, Thermo Fisher Scientific).

### EV Treatment

All mouse work was performed in accordance with national animal experimentation guidelines (DGAV), animal protocol 0421/000/000/2018. Adult C57Bl/6 female mice (5 to 8 weeks old) were used for all experimental procedures. Mice were anesthetized using isoflurane 1.5–3%. Five micrograms of EVs were injected into the retro-orbital venous sinus in a total volume of 100 μl filtered PBS. For education experiments, mice received 5 μg of EVs every other day, three times per week for 3 weeks. In the experiments involving evaluation of EV incorporation, labeled EVs were injected 24 h prior to tissue collection, and analysis of EV^+^ cells was performed by flow cytometry or immunofluorescence. Unlabeled EVs were used as controls for signal specificity. For education experiments, retro-orbital injection of PBS was used in the control groups.

### Flow Cytometry Analysis

For tracking of labeled EVs and phenotypical analysis of murine organs, femurs were flushed and single-cell suspensions were filtered through a 40-μm strainer. Cells were washed in PBS with 1% BSA and incubated with anti-CD11b-PerCP-Cyanine5.5 (clone M1/70, 1:100, BD Biosciences – 561114) at predetermined saturating concentrations. PKH67-labeled EV-positive cells were detected using blue laser excitation and 488 nm emission. Data for 1,000,000 cells was acquired on a BD FACS Canto^TM^ cytometer with Diva software (BD) and was analyzed using FlowJo^TM^ software (TreeStar).

### Collection of BM-Derived Cells and CD11b^+^ Magnetic Sorting

C57Bl/6 female mice were euthanized and femurs and tibiae were harvested and cleaned. The bones were flushed with cold working buffer (PBS supplemented with 0.5% BSA and 2 mM EDTA), using a 26G needle. The preparation was resuspended using a 18G needle and filtered through a 40-μm strainer. The single-cell preparation was then centrifuged (500 *g*, 10 min, 4°C) and the resulting pellet resuspended in ACK buffer. After 2 min at room temperature, the lysis was stopped by adding more working buffer and the sample was centrifuged (500 *g*, 10 min, 4°C). The pellet was resuspended in working buffer and the cells were counted.

Subsequently, cells underwent MACS bead isolation using CD11b microbeads (Miltenyi Biotec, 130-049-601, Germany), which enabled the isolation of CD11b^+^ cells from the preparation. The CD11b^+^ cells were counted and centrifuged (500 *g*, 10 min, 4°C) and the pellet proceeded for RNA isolation.

### RNA Extraction and cDNA Preparation

Prior to RNA extraction, CD11b^+^ BM cells were run through the QIAshredder kit (79654, Qiagen, Germany). Total RNA was isolated using the RNeasy Mini kit (74104, Qiagen), following the manufacturer’s instructions, and resuspended in nuclease-free water. RNA concentration and purity were estimated by spectrophotometric absorbance (260 and 280 nm) using a Nanodrop 2000 unit (Thermo Scientific). One microgram of RNA was used to prepare cDNA using the QuantiTect Reverse Transcription Kit (205311, Qiagen), according to the manufacturer’s instructions.

### Illumina Strand-Specific RNA Sequencing

To analyze the genes whose expression was altered in mouse CD11b^+^ BM cells as a result of education with PAN02 EVs, animals were grouped between the ones educated with PAN02 EVs and the control animals educated with PBS. Total RNA was then isolated as described above, and the RNA quality was assessed using Bioanalyzer. The cDNA library was generated using Kappa Stranded Total RNA with Ribo-Zero Library Preparation Kit. The resultant DNA fragments (DNA library) were sequenced in the Illumina HiSeq 4000 platform, using 150 bp paired-end sequencing reads.

For the analysis of differentially expressed genes, we used the Differential Expression for RNA-Seq tool, which is a multifactorial statistical analysis tool based on a negative binomial model. It uses a generalized linear model approach influenced by the multifactorial EdgeR method (Robinson et al., 2009). The differentially expressed genes were filtered using standard conditions (van Iterson et al., 2010; Raza and Mishra, 2012), and the genes that fulfilled both conditions were listed in the results (Supplementary Table 1). The conditions were as follows: fold change (≥ 2 or ≤ −2) and false discovery rate (FDR) *P*-value ≤ 0.05.

### Primer Design

The primers used in this study were designed using Integrated DNA Technologies online software PrimerQuest Tool (Integrated DNA Technologies, Inc., United States). Primer sequences and characteristics are shown in Supplementary Table 2.

The amplification efficiencies of each selected gene performing quantitative RT-PCR (RT-qPCR) were evaluated using cDNA dilutions (1, 1:10, 1:100, 1:1,000, 1:10,000). The amplification efficiency *E* of all primers used was measured and all displayed high *E*-values ranging from 1.8 to 2.2 (Supplementary Table 2).

### RT-qPCR Assay and Analysis

The qRT-PCR reactions were performed in a CFX96 Touch Real-Time PCR Detection System thermocycler (Bio-Rad, United States). The reaction mix was performed using 10 μl SsoFast EvaGreen Supermix (1725200, Bio-Rad), primers at a final concentration of 500 nM, 1 μl of cDNA, and nuclease-free water to complete a final volume of 20 μl. After PCR, a melting curve program from 65 to 95°C with 0.5°C changes was applied, and the presence of a single reaction product in each well was confirmed. All reactions were performed in duplicate and technical replicates were run on the same plate. For the analysis, the threshold value used for each plate was the one defined by the software.

The relative expression was calculated using the model proposed by M.W. Pfaffl (2001) and normalized to both *Gapdh* and *Hmbs* levels, the two reference genes used.

### Mass Spectrometry

#### Peptide Sample Preparation

The protein solution containing SDS and dithiothreitol (DTT) was loaded onto filtering columns and washed exhaustively with 8 M urea in HEPES buffer (Wisniewski et al., 2009). Proteins were reduced with DTT and alkylated with IAA. Protein digestion was performed by overnight digestion with trypsin sequencing grade (Promega).

#### Nano-LC-MSMS Analysis

Peptide samples were analyzed by nano-LC-MSMS (Dionex RSLCnano 3000) coupled to a Q-Exactive Orbitrap mass spectrometer (Thermo Scientific). Briefly, the samples (5 μl) were loaded onto a custom-made fused capillary precolumn (2 cm length, 360 μm OD, 75 μm ID) with a flow of 5 μl per min for 7 min. Trapped peptides were separated on a custom-made fused capillary column (20 cm length, 360 μm outer diameter, 75 μm inner diameter) packed with ReproSil-Pur C18 3-μm resin (Dr. Maish, Ammerbuch-Entringen, Germany) with a flow of 300 nl per minute using a linear gradient from 92% A (0.1% formic acid) to 28% B (0.1% formic acid in 100 acetonitrile) over 93 min followed by a linear gradient from 28 to 35% B over 20 min at a flow rate of 300 nl per minute. Mass spectra were acquired in positive ion mode applying automatic data-dependent switch between one Orbitrap survey MS scan in the mass range of 400 to 1,200 *m*/*z* followed by higher-energy collisional dissociation (HCD) fragmentation and Orbitrap detection of the 15 most intense ions observed in the MS scan. Target value in the Orbitrap for MS scan was 1,000,000 ions at a resolution of 70,000 at *m*/*z* 200. Fragmentation in the HCD cell was performed at normalized collision energy of 31 eV. Ion selection threshold was set to 25,000 counts and maximum injection time was 100 ms for MS scans and 300 and 500 ms for MSMS scans. Selected sequenced ions were dynamically excluded for 45 s.

#### Database Search

The obtained data from the X LC-MS runs were searched using VEMS (Matthiesen et al., 2012; Carvalho et al., 2014) and MaxQuant (Cox and Mann, 2008). A standard proteome database from UniProt (3AUP000005640), in which common contaminants were included, was also searched. Trypsin cleavage allowing a maximum of four missed cleavages was used. Carbamidomethyl cysteine was included as fixed modification. Methionine oxidation, N-terminal protein acetylation, and S, T, and Y phosphorylation were included as variable modifications; 5 ppm mass accuracy was specified for precursor ions and 0.01 *m*/*z* for fragment ions. The FDR for protein identification was set to 1% for peptide and protein identifications. No restriction was applied for minimal peptide length for VEMS search. Identified proteins were divided into evidence groups as defined (Matthiesen et al., 2012).

### Negative-Staining Transmission Electron Microscopy

Extracellular vesicles were visualized by transmission electron microscopy (TEM) using negative staining. For this, 10 μl of the sample solution was mounted on formvar/carbon film-coated mesh nickel grids (Electron Microscopy Sciences, Hatfield, PA, United States). The excess liquid was removed with filter paper, and 10 μl of 1% uranyl acetate was added onto the grids. Visualization was carried out on a JEOL JEM 1400 TEM at 120 kV (Tokyo, Japan). Images were digitally recorded using a CCD digital camera (Orious 1100W Tokyo, Japan).

### Statistical and Pathway Analysis

Error bars in graphical data represent means ± SEM. Statistical significance was determined using a two-tailed Student’s *t*-test or by ANOVA. *P* < 0.05 was considered statistically significant. Statistical analyses were performed using the GraphPad Prism software (GraphPad software). No statistical method was used to predetermine sample size. The experiments were not randomized, and the investigators were not blinded to allocation during the experiments and outcome assessment.

### Accession Codes

The raw sequencing data (Supplementary Table 1) have been deposited in the GEO database under accession number GSE156071.

## Results

**FIGURE 1:**
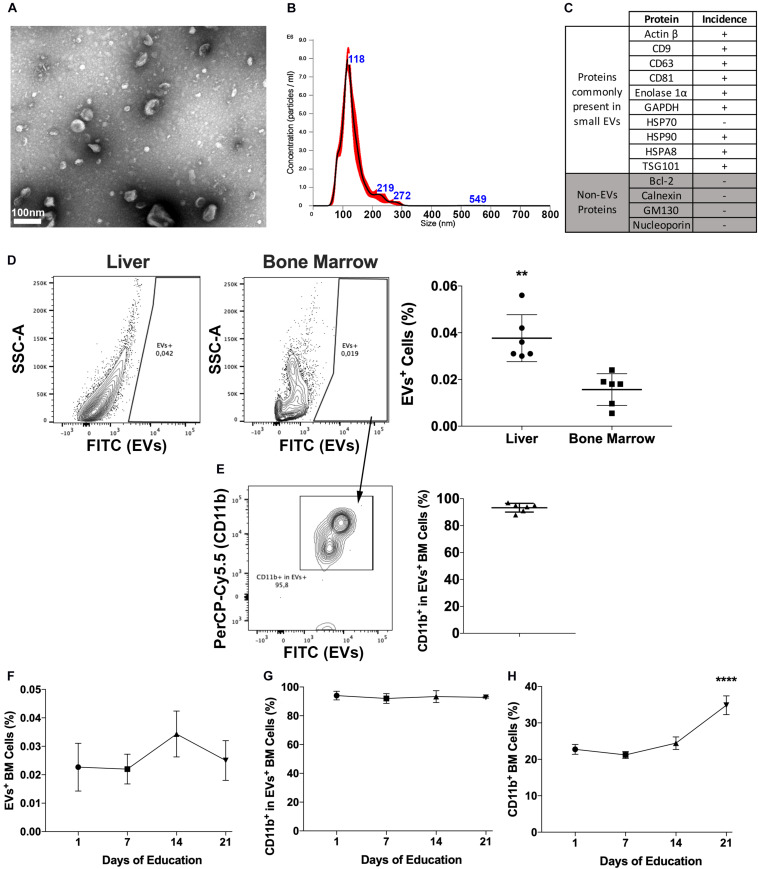
Pancreatic cancers-derived EVs binding to BM cells. **(A)** Representative transmission electron microscopy of PAN02 EVs. **(B)** Representative Nanoparticle Tracking Analysis of PAN02 EVs. **(C)** Proteins frequently present and absent in small EVs, studied in PAN02 EVs by Protein Mass Spectrometry. **(D)** PC-EVs are taken up by liver and BM cells, 24 h post-injection. **(E)** Most of the cells that take up PC-EVs are CD11b^+^ BM cells. **(F)** Three-week education with PC-EVs does not modify the incidence of EV^+^ BM cells or CD11b^+^ cells within cells that take up PC-EVs **(G)**. **(H)** Three-week education with PC-EVs induces the increase of BM CD11b^+^ cells. **P < 0.01, ****P < 0.0001 by ANOVA.

Isolated EVs were characterized for morphology and size by transmission electron microscopy (Figure 1A) and for size distribution by Nanoparticle Tracking Analysis (Figure 1B) and the expression of proteins commonly identified in EVs (Figure 1C). Currently, there are no available methods to isolate EVs expressing endosomal features and consensus on markers that could be used to differentiate endosomal (i.e., exosomes) from membrane shedding-derived vesicles (i.e., microvesicles). In fact, even molecules considered as markers of small endosomal EVs, such as HSP70, CD63, and CD9, have been reported to be present both in small and large EVs (Kowal et al., 2016). Therefore, although the majority of our vesicles display exosome features, including size (Figures 1A,B) and molecular composition (Figure 1C and Supplementary Table 3), we decided to identify them as EVs throughout the manuscript to avoid potential sample misidentification, following the latest MISEV’s recommendations (Thery et al., 2018).

To first determine the occurrence of PC–BM interaction, labeled PC-EVs were injected intravenously in mice. Besides the liver, 24-h post-injection EVs were also located in the BM, albeit the percentage of EVs^+^ cells in the BM was lower (∼0.02%) than the one found in the liver (∼0.04%) (Figure 1D). In the BM, more than 80% of the cells that took up PC-EVs were CD11b^+^ (Figure 1E). To our knowledge, this is the first report of a direct PC–BM communication axis mediated by EVs.

To evaluate if this proportion increased over time and upon continuous treatment, animals were also educated (3 weeks, every other day) with PC-EVs. No differences in the percentage of EVs^+^ BM cells were found throughout the experiment (Figure 1F). The percentage of CD11b^+^ cells among EVs^+^ BM cells also did not oscillate throughout the experiment (Figure 1G). However, education with PC-EVs increased the percentage of CD11b^+^ BM cells at the experiment endpoint (Figure 1H).

**FIGURE 2:**
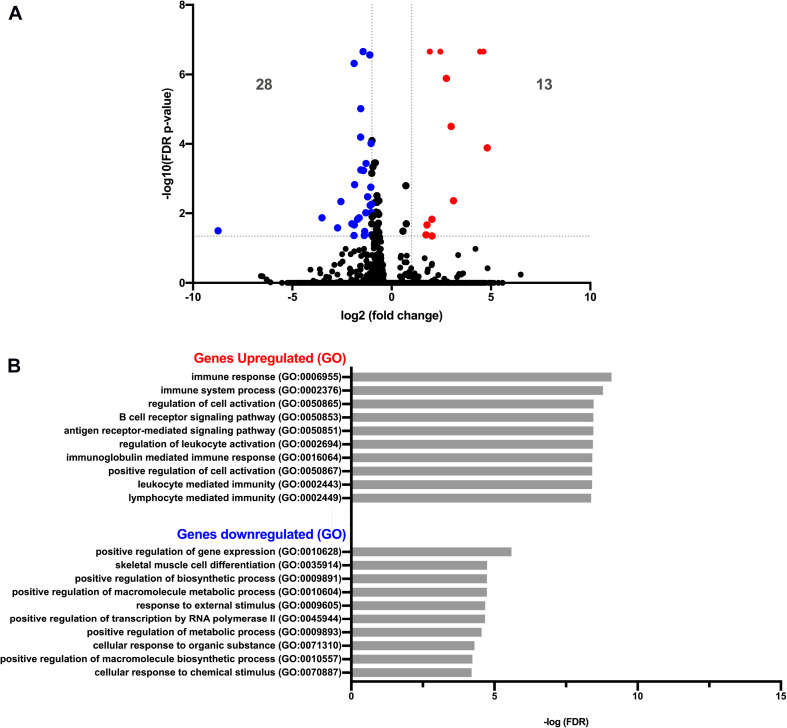
Gene expression analysis of pancreatic cancer EV-treated bone marrow cells. **(A)** Volcano plot of RNA-Seq of PBS versus EV-treated CD11b^+^ BM cells showing 13 and 28 genes significantly up- and downregulated (red dots and blue dots, respectively), with FDR *P*-value ≤ 0.05 and fold change > 2 or < −2. **(B)** Graph plot of the most significant processes of Gene Ontology (GO) enrichment analysis in the “Biological Process” section of up- and downregulated genes, using the Gene Ontology PANTHER Database.

We next asked whether PC-EVs can modify the gene expression profile of CD11b^+^ BM cells. For that, we sequenced RNA samples extracted from CD11b^+^ BM cells of both naive and PC-EV-educated mice. The expression levels of all samples were assessed through the mapping of the high-quality reads of each sample, where 88.9 to 94.38% of the total fragments were mapped against the *Mus musculus* (GRCm38) genome. The differentially expressed genes were selected using standard conditions (FDR *P*-value ≤ 0.05 and fold change > 2 or < −2), which yielded a total of 41 genes (Figure 2A). Of these, 13 genes were significantly upregulated (Table 1) and 28 significantly downregulated (Table 2). These results were validated by qPCR analysis of two of the top differentially expressed genes (Supplementary Figure 1).

**TABLE 1:** Upregulated genes.

Name	FDR *p*-value	Log*2* fold change	ENSEMBL
Immunoglobulin heavy variable 1-26 (Fragment)	0	4,623730851	ENSMUSG00000094546
Immunoglobulin kappa variable 4-59 (Fragment)	0	4,447764161	ENSMUSG00000094006
Immunoglobulin J chain	0	2,449246148	ENSMUSG00000067149
Immunoglobulin heavy constant alpha (Fragment)	0	1,927167042	ENSMUSG00000095079
Immunoglobulin kappa constant (Fragment)	0	1,914161392	ENSMUSG00000076609
Ig gamma-1 chain C region secreted form (Fragment)	1,30134E-06	2,752343692	ENSMUSG00000076614
Predicted gene, 49345 (Fragment)	3,1653E-05	2,99697945	ENSMUSG00000114923
Immunoglobulin heavy variable V3-8 (Fragment)	0,000129751	4,808095452	ENSMUSG00000076674
Immunoglobulin kappa variable 1-110 (Fragment)	0,004373421	3,110850502	ENSMUSG00000093861
U3A small nuclear RNA	0,014852817	2,027515231	ENSMUSG00000106147
Immunoglobulin lambda constant 1 (Fragment)	0,021654529	1,776317776	ENSMUSG00000105906
Immunoglobulin lambda variable 1 (Fragment)	0,041671894	1,728395758	ENSMUSG00000076934
Immunoglobulin heavy constant gamma 3 (Fragment)	0,044897113	2,031085382	ENSMUSG00000076615

**TABLE 2:** Downregulated genes.

Name	FDR *p*-value	Log*2* fold change	ENSEMBL
Nuclear receptor subfamily 4 group A member 1	2,19745E-07	−1,435869584	ENSM USG 00000023034
Lipoprotein lipase	2,74851E-07	−1,09850161	ENSMUSG00000015568
Cyclic AMP-dependent transcription factor ATF-3	4,81325E-07	−1,887929851	ENSM USG 00000026628
ATP synthase protein 8	9,71391E-06	−1,554520723	ENSM USG 00000064356
Early growth response protein 3	6,39601E-05	−1,565012389	ENSM USG 00000033730
Krueppel-like factor 4	9,72554E-05	−1,036425344	ENSM USG 00000003032
Early growth response protein 1	0,000369143	−1,293024511	ENSMUSG00000038418
Prostaglandin G/H synthase 2	0,000573753	−1,548440352	ENSM USG 00000032487
Probable leucine–tRNA ligase, mitochondrial	0,000590408	−1,422122013	ENSM USG 00000035202
Regulator of G-protein signaling 1	0,001504139	−1,862241816	ENSM USG 00000026358
Monocyte differentiation antigen CD14	0,001783497	−1,048560286	ENSMUSG00000051439
C-C motif chemokine 3	0,003368206	−1,210768022	ENSM USG 00000000982
Predicted gene, 47075	0,0046162	−2,554952242	ENSMUSG00000114169
Cyclin-dependent kinase inhibitor 1	0,005512827	−1,012425917	ENSM USG 00000023067
Proto-oncogene c-Fos	0,005961159	−1,078598039	ENSMUSG00000021250
Transcription factor Spi-C	0,009588443	−1,02746831	ENSM USG 00000004359
Fos-related antigen 1	0,009676491	−1,304846194	ENSMUSG00000024912
Nuclear receptor subfamily 4 group A member 2	0,013476224	−1,63691541	ENSM USG 00000026826
Predicted gene, 23262	0,013476224	−3,507668615	ENSM USG 00000088948
Predicted gene 6377	0,014852817	−1,716519183	ENSM USG 00000048621
X-linked lymphocyte-regulated protein PM1	0,020080701	−1,997616005	ENSM USG 00000054626
C-C motif chemokine 2	0,021654529	−1,882312446	ENSM USG 00000035385
Predicted gene, 47088	0,026317916	−2,725652374	ENSMUSG00000113076
Predicted gene, 48275	0,031964977	−8,738521219	ENSMUSG00000111202
E3 SUMO-protein ligase EGR2	0,033340755	−1,358834793	ENSM USG 00000037868
Hematopoietic prostaglandin D synthase	0,041671894	−1,012816032	ENSMUSG00000029919
Predicted gene 45053	0,043671552	−1,376757203	ENSMUSG00000108368
Mitochondrially encoded 16S rRNA	0,043671552	−1,896767497	ENSM USG 00000064339

Gene Ontology (GO) analysis revealed that the upregulated genes (Table 1) were associated with immune response processes and immunoglobulin production (Figure 2A). Among those genes, we found the Immunoglobulin heavy constant gamma 3 (fragment) (*Ighg3*) gene, which is associated with the IgG3 isotype, and the genes Immunoglobulin heavy constant alpha (fragment) (*Igha*) and Immunoglobulin J chain (*Jchain*), both associated with IgA molecules. Within the downregulated genes, we found that the most significant GO processes were associated with transcriptional activation, response to stimulus, and regulation of gene expression (Figure 2B). Specifically, most downregulated genes were transcription factors associated with monocyte and macrophage differentiation, macrophage polarization, production of pro- or anti-inflammatory cytokines, and immune cell trafficking (Table 2). Interestingly, the majority of downregulated genes are relevant in the tumor microenvironment context.

## Discussion

In the present study, we aimed to identify a novel systemic cell–cell communication axis with potential relevance to tumor microenvironment dynamics. Previously, we have demonstrated that PC-derived EVs can induce a liver microenvironment supportive of tumor development and metastasis, LPMN, by acting in non-tumor cells in the liver. The LPMN cascade of events involves the accumulation of CD11b^+^ BM cells in the liver, which is associated with the formation of hepatic PC metastatic lesions (Costa-Silva et al., 2015). For this reason, we asked if EVs could also mediate a direct communication between PC and CD11b^+^ BM cells *in vivo*. We found that PC-EVs bind preferentially to CD11b^+^ BM cells and induce the expansion of this cell population upon 3 weeks of treatment, every other day with PC-EVs. These results agree with previous reports of EVs mediating the communication of other cancer types and BM (Yu et al., 2007; Peinado et al., 2012).

We next showed that PC-EVs can reprogram the gene expression of recipient CD11b^+^ BM cells. Indeed, various studies describe tumor EVs as capable of impacting myeloid cell function (Valenti et al., 2006; Yu et al., 2007; Shen et al., 2017) whereas PC-EVs were shown to regulate the expression of TLR4 in dendritic cells *in vitro* (Zhou et al., 2014). EVs carry all main biomolecules, including lipids, metabolites, proteins, and/or nucleic acids, which can impact the recipient cells and mediate several biological processes such as tumor growth and invasion, inflammation, and immunologic remodeling. Various studies describe the role of EV lipids, namely PGE2, in tumor growth and resistance (Record et al., 2018). The role of metabolites transported by EVs cannot be dismissed since EVs derived from MSCs are packaged with numerous metabolites that have been directly associated with immunomodulation, including M2 macrophage polarization and regulatory T lymphocyte induction (Showalter et al., 2019). Worth mentioning is also the role of microRNA within EVs, since mir-145 can regulate the polarization of tumor-associated macrophages (Shinohara et al., 2017). In the past, we also described the relevance of the protein content in tumor EV bioactivity, when we described that once taken up by hepatic resident macrophages, in a mechanism mediated by EV Integrin αVβ5 (Hoshino et al., 2015), pancreatic cancer-derived EVs containing high levels of MIF (Costa-Silva et al., 2015) induced upregulation of secreted factors associated with liver fibrosis, such as TGF-β (Costa-Silva et al., 2015), and pro-inflammatory genes involved with metastasis, such as S100A8 and S100P (Lukanidin and Sleeman, 2012; Hoshino et al., 2015). Considering the literature on this topic, we expect that the response of CD11b BM cells to PC-EVs described in this manuscript most likely involves numerous molecules and different pathways, suggesting that it could be simultaneously mediated by multiple EV biomolecules. Our work did not evaluate *in vivo* the potential role of the genes differentially expressed in BM CD11b^+^ cells upon PC-EV education in the setup of prometastasis hepatic niches. Therefore, follow-up *in vivo* studies will be necessary to test the potential role of each of the identified genes in PC liver metastasis. Future investigation will enable the identification of which EV biomolecules mediate the transcription reprogramming here described. In addition, based on previous works that utilized plasma EVs as biomarkers for diagnosis, prognosis, and follow-up of cancer patients (Peinado et al., 2012; Costa-Silva et al., 2015; Hoshino et al., 2015, 2020; Melo et al., 2015), it is possible that EV biomolecules linked with PC-CD11b^+^ BM cell communication may enable early detection of this process through the study of circulating plasma EVs. We will here discuss on genes regulated by PC-EVs with potential relevance to tumor microenvironments and the biology of monocytes/macrophages.

### Immunoglobulin Genes

The production of immunoglobulins has been classically linked to lymphoid B cells. However, recent evidence challenged the existing dogma by showing expression of immunoglobulins outside the lymphoid lineage (e.g., monocytes and macrophages) in oncologic settings (Haziot et al., 2018; Busch et al., 2019). These works describe immunoglobulin expression as a defining feature of monocytes and also macrophages in the tumor microenvironment.

Increased levels of immunoglobulin expression in tumor microenvironments are described to result in the accumulation of immune complexes that favor tumor-promoting inflammatory responses, including recruitment and activation of several myeloid cell types (Barbera-Guillem et al., 1999). Both IgG and IgA are relevant in the tumor microenvironment, having anti- and protumoral effects. IgG is described to promote cell growth and metastasis, inhibit apoptosis, and play a role in cancer immune evasion (Wan et al., 2015; Xu et al., 2016). Synergistically, IgG and tumor cell-derived debris are able to promote metastasis of pancreatic cancer by inducing inflammation *via* M2-polarized tumor-associated macrophages (TAMs) (Chen et al., 2019). Moreover, IgGs have been shown to promote cancer development by activating Fc-γ receptors on resident and recruited leukocytes that in turn regulate recruitment, composition, and effector functions of tumor-infiltrating lymphocytes in a mouse model of squamous carcinogenesis (Andreu et al., 2010). On the other hand, some studies show IgG1 supporting immunity against tumors, inducing antibody-dependent cell-mediated cytotoxicity (ADCC) and antibody-dependent cellular phagocytosis (ADCP) (Kellner et al., 2017).

It has also been shown that IgA can block cytotoxic T-cell reactions against melanoma (O’Neill and Romsdahl, 2009). More recently, IgA^+^ plasmocytes were shown to suppress antitumor immunity in a mouse prostate cancer model. However, in this study, it is unclear if the suppression can be attributed to IgA^+^ plasmocytes or IgA alone (Shalapour et al., 2015). By contrast, IgA shows a therapeutic potential against cancer cells, since it activates neutrophil-mediated antibody-dependent cellular cytotoxicity better than IgG (Frontera et al., 2018; Steffen et al., 2020). It should be highlighted though that confirmation that immunoglobulins produced by non-lymphoid cells are functional is still pending, since studies on whether affinity-matured antibodies from these cells are capable of binding to antigens are not yet available. Additionally, we cannot exclude the potential presence of B1 cells, a small immunoglobulin-expressing phagocytic population that is believed to originate from B cells, within our CD11b^+^ BM cells (Griffin and Rothstein, 2011).

In light of these findings, and taking into consideration the previously described direct association between CD11b^+^ BM cell accumulation in the liver and the formation of hepatic PC metastatic lesions, our results suggest that PC-EV-induced immunoglobulin expression on CD11b^+^ BM cells may play a role in PC liver metastasis. Nevertheless, it is still unclear which specific epitopes are recognized by these antibodies and whether they contribute to tumor microenvironments (Haziot et al., 2018; Busch et al., 2019).

### Genes Linked With Monocyte/Macrophage Differentiation

Among the downregulated genes identified, lipoprotein lipase (*Lpl*) (Chang et al., 2018), transcription factor Spi-c (*Spic*) (Kohyama et al., 2008; Haldar et al., 2014; Alam et al., 2017; Kurotaki et al., 2017), and Nuclear receptor subfamily 4 group A member1 (*Nr4a1*) (Hanna et al., 2011) have all been previously found to induce differentiation of monocytes. Another gene potentially linked with monocyte/macrophage differentiation is CDK inhibitor 1, which when downregulated leads to higher expression of CD11b, thus modifying cellular differentiation (Radomska et al., 2012; McClure et al., 2016). Other downregulated genes, early growth response protein 1 (*Egr1*) and 3 (*Egr3*), were believed to have an essential role in regulating monocyte/macrophage differentiation, but were later suggested to have no impact in the macrophage differentiation process (Carter and Tourtellotte, 2007). Hence, our results suggest a mixed effect of PC-EVs in CD11b^+^ BM cell differentiation, with some of the downregulated genes found having a positive impact on the differentiation process and others having a negative impact in the same process. A more detailed functional characterization will thus be necessary to understand the final balance of the downregulation of these genes by PC-EVs in CD11b^+^ BM cell differentiation, as well as their role in PC.

### Genes Linked With Tumor Immunity

In the establishment of tumor-supportive microenvironments, macrophage polarization is pivotal. Reduced expression of Cyclic AMP-dependent transcription factor ATF-3 (*Atf3*) (Labzin et al., 2015; Sha et al., 2017), Fos-related antigen 1 (*Fosl1*) (de Marcken et al., 2019), and *Nr4a1* (Hamers et al., 2016) can impact pro-inflammatory M1 polarization and antitumor immunity (Kratochvill et al., 2015; Chiba et al., 2018), as they negatively regulate these processes. C-C chemokine ligand 3 (*Ccl3*) (Cassol et al., 2009) was also reported to be involved in the M1 macrophage polarization and CD14 was shown not only to contribute to a M1 phenotype but also to drive strong Th1/Th17 signaling in macrophages and circulating dendritic cells (Prakash et al., 2016). Reduced expression of the Proto-oncogene c-Fos (*c-fos*) and *Egr1* is expected to reduce the expression of pro-inflammatory and antitumoral cytokines, which are also involved in the M1 polarization (Krämer et al., 1994; Ray et al., 2006; Maruyama et al., 2007; Cullen et al., 2010; Hop et al., 2018; Yu et al., 2018). Conversely, reduced expression of *Atf3* (Sha et al., 2017), *Klf4* (Ahmad et al., 2018; Wen et al., 2019), and Prostaglandin G/H synthase 2 (*Pghs-2*) (Wang X. et al., 2017) in macrophages can favor a pro-inflammatory M1 polarization, hence potentially favoring antitumoral responses.

On the other hand, some of the genes downregulated by PC-EVs were also found to support tumor development, as C-C chemokine ligand 2 (*Ccl2*) (Roca et al., 2009; Li et al., 2017), *Atf3* (Sha et al., 2017), Hematopoietic prostaglandin D synthase (Abbas and Janeway, 2000), Krüppel-like factor 4 (*Klf4*) (Kapoor et al., 2015; Wang K. et al., 2017), Nuclear receptor subfamily 4 group A member 2 (*Nr4a2*) (Mahajan et al., 2015), and *Pghs-2* (Li et al., 2015) were linked to M2 polarization of macrophages. The *Egr2* gene has been described as a M2 macrophage marker and it has been implicated in fate determination in the myeloid lineage (Gabet et al., 2010; Jablonski et al., 2015). Specifically, an *Egr2* knockdown model failed to show upregulation of either M1 or M2 markers upon stimulation, and low levels of *Egr2* expression were associated with non-responsiveness of macrophages to activation signals (Veremeyko et al., 2018).

Besides the discussed effects on M1/M2 macrophage polarization, several of the downregulated genes were associated with other antitumoral effects, which may lead to a broader range of protumorigenic outcomes. That is the case of *Nr4a1*, *Ccl2*, and *Ccl3*, which antagonize tumor growth by attracting tumor-suppressive immune cells. Indeed, infiltration of inflammatory cells in the tumors of *Nr4a1*^–/–^ mice was diminished when compared with *Nr4a1*^+/+^ mice (Ahmad et al., 2017), and the inhibition of *Ccl2* promoted neocarcinogenesis and metastasis (Huang et al., 1994; Li et al., 2014; Lavender et al., 2017). *Ccl2* (Granot et al., 2011; Mitchem and DeNardo, 2012) has also been reported to increase the cytotoxicity of neutrophils against tumor cells. Thus, reduction of *Ccl2* expression can decrease neutrophil-mediated killing of tumor cells and promote a protumorigenic microenvironment.

We here suggest that the downregulation of these genes by PC-EVs could induce a combined anti-/protumorigenic response by BM CD11b^+^ cells. Additional *in vivo* studies will be necessary to evaluate whether these inflammatory profiles will be reversed and/or reinforced after the influx of these cells in the liver, where Kupffer cell-derived TGF-β (Costa-Silva et al., 2015) can play a potential role in inducing protumorigenic genes (Gratchev, 2017) by BM-derived CD11b^+^ cells.

### Genes Linked With Monocyte/Macrophage Trafficking

Genes associated with monocyte and macrophage recruitment, such as *Ccl2* (Qian et al., 2011; Carson et al., 2017; Gschwandtner et al., 2019), *Ccl3* (Ntanasis-Stathopoulos et al., 2020), and *Fosl1* (Jiang et al., 2019), were found to be downregulated. The decrease in the levels of *Fosl1*, *Ccl2*, and *Ccl3* is expected to reduce the recruitment of cells that allow tumor cells to evade the immune surveillance, e.g., TAMs, regulatory T cells (Tregs), and myeloid-derived suppressor cells (MDSCs) (Jiang et al., 2019; Wculek et al., 2019; Ntanasis-Stathopoulos et al., 2020). Furthermore, downregulated *Ccl3* could reduce the recruitment of cytotoxic neutrophils, dendritic cells, and NK cells; reduce CD8^+^ antitumor response; and lead to impaired antigen presentation, reduced levels of IFN-γ by antigen-specific T cells, and consequently, reduced cytotoxic activity (Song et al., 2007; Ntanasis-Stathopoulos et al., 2020). Overall, a decrease in the recruitment of monocytes and macrophages could shape the tumor microenvironment toward a less supportive setting for cancer progression.

Another downregulated gene, *Atf3*, has been linked with increased intrahepatic macrophage/neutrophil trafficking (Zhu et al., 2018). Regulator of G-protein signaling 1 (*Rgs1*), also downregulated by PC-EVs, has been involved in the chemoattraction of monocytes. Specifically, *Rgs1* deactivates the chemotactic response (Denecke et al., 1999), and its deficiency leads to enhanced recruitment of macrophages (Patel et al., 2015). We speculate that the *Rgs1* downregulation in CD11b^+^ BM cells can lead to less myeloid accumulation and more monocyte/macrophage trafficking.

In most of these studies, genes were downregulated in the whole animal, instead of only in BM cells. Therefore, more detailed *in vivo* experiments would be needed to test whether reduced expression of these genes in CD11b^+^ BM cells could impact their trafficking to PC microenvironments.

### Genes Linked With Monocyte/Macrophage Apoptosis

The downregulation of *Ccl2* (Salcedo et al., 2000; Lavender et al., 2017), *Fosl1* (Lewçn et al., 2007; Luo et al., 2009), and *Rgs1* (Hu et al., 2019) has been described to increase apoptosis and to decrease angiogenesis and tumor invasion by affecting molecules associated with these processes (e.g., VEGF, MMP-2, MMP-9). On the other hand, the downregulated genes *Egr1* (Chen et al., 2010) and *Atf3* (Wong, 2011) have been described as proapoptotic, and thus, the decrease in their expression alone might provide a higher degree of protection against apoptosis in CD11b^+^ BM cells.

## Conclusion

We here found a novel PC–BM communication axis mediated by PC-EVs. Our gene expression analysis showed reprogramming of genes linked to both anti- and protumorigenic activities by CD11b^+^ cells. Their potential relevance in PC tumor microenvironments was discussed, although how the individual effects of the reprogrammed genes balance each other to a final anti- or protumorigenic effect is currently unclear. Future *in vivo* studies involving single-cell transcriptome and detailed analysis of the role of each gene in the phenotype of CD11b^+^ BM cells will be necessary to clarify whether and how PC-EVs uptake by these cells contributes to the setup of PC microenvironments. Stepping further into the future, the study of PC-educated BM-derived cells linked with PC metastasis in the peripheral blood could be used as a minimally invasive method to detect and monitor these protumorigenic niches in clinical settings, thus having a potential impact in improving early diagnosis, treatment, and follow-up of patients with PC and other oncologic diseases. Additionally, it has the potential to contribute to the development of therapeutic targeting of BM-derived cells that promote liver metastatic disease.

## Data Availability Statement

The datasets presented in this study can be found in online repositories. The names of the repository/repositories and accession number(s) can be found in the article/ Supplementary Material.

## Ethics Statement

All mouse work was performed in accordance with national animal experimentation guidelines (DGAV), animal protocol 0421/000/000/2018.

## Author Contributions

JM designed and performed the experiments. AM and JP performed the transmission electron microscopy studies. AO, AC, RM, and HB performed the mass spectrometry studies. MS built the experimental setup. BC-S conceived the project. All authors wrote and reviewed the manuscript.

## Conflict of Interest

The authors declare that the research was conducted in the absence of any commercial or financial relationships that could be construed as a potential conflict of interest.

## Supplementary Material

Supplementary Figure 1 Validation of differentially expressed genes by quantitative PCR (qPCR). Depicted in the graph is the fold change of CD11b^+^ BM cells from animals treated PC EVs or PBS (control). Ighv1-26 and Ighv4-59 relative gene expression was evaluated by qPCR. Error bars represent ± SEM for *n* = 4, each point represents a pool of at least 5 animals. qPCR samples were normalized to both reference genes (*Gapdh* and *Hmbs*).
