# Supplementary figures and images for: Transcriptome Reprogramming of CD11b+ Bone Marrow Cells by Pancreatic Cancer Extracellular Vesicles
Source: Front Cell Dev Biol. 2020 Nov 27;8:592518. doi: 10.3389/fcell.2020.592518 (PMC7729189; doi:10.3389/fcell.2020.592518)

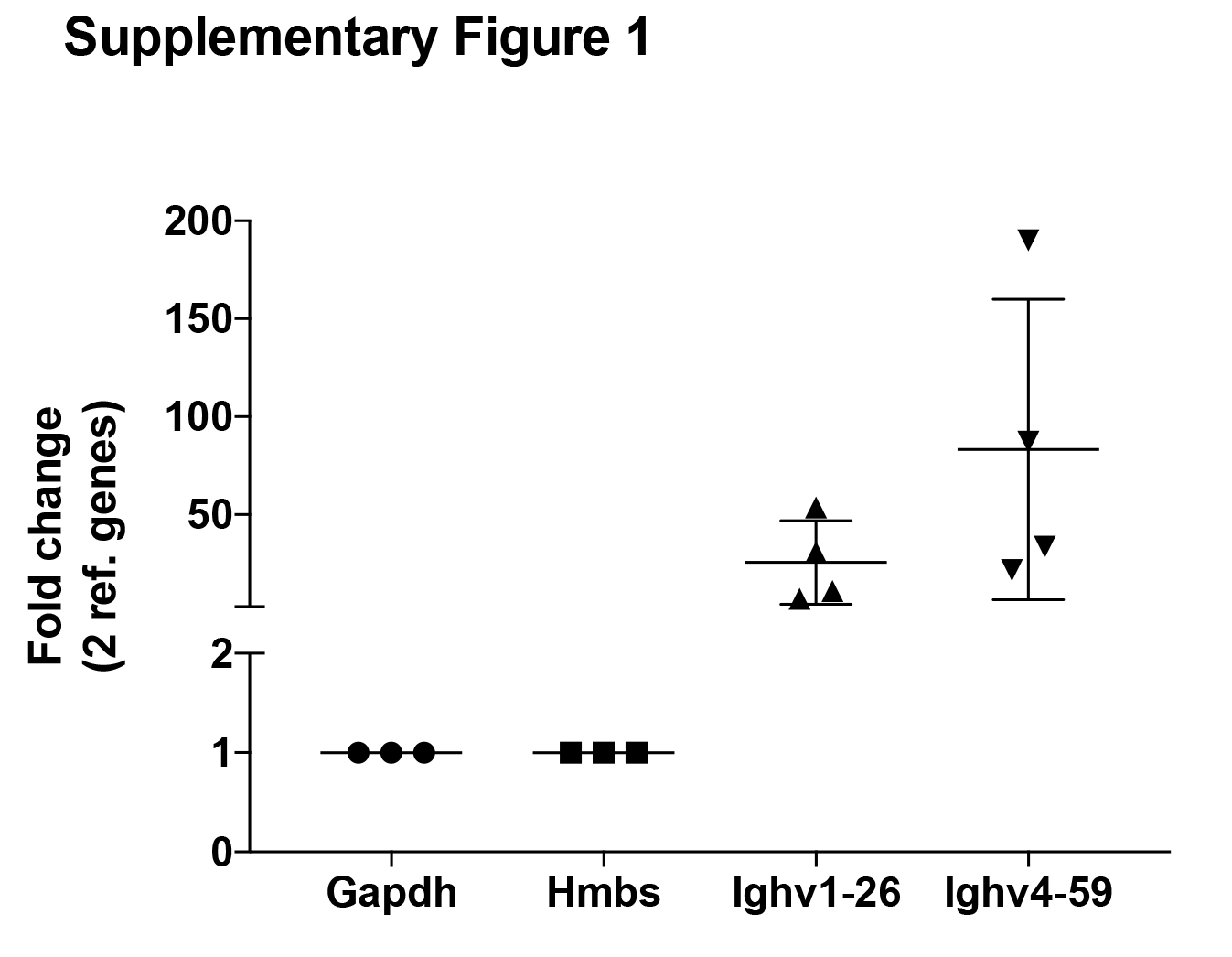

Supplement: Supplementary Figure 1 — Validation of differentially expressed genes by quantitative PCR (qPCR). Depicted in the graph is the fold change of CD11b+ BM cells from animals treated PC EVs or PBS (control). Ighv1-26 and Ighv4-59 relative gene expression was evaluated by qPCR. Error bars represent ± SEM for n = 4, each point represents a pool of at least 5 animals. qPCR samples were normalized to both reference genes (Gapdh and Hmbs). [file Image_1.tiff]
